# Alleviating the Effect of Branched-Chain Fatty Acids on the Lipopolysaccharide-Induced Inflammatory Response in Calf Small Intestinal Epithelial Cells

**DOI:** 10.3390/antiox14050608

**Published:** 2025-05-19

**Authors:** Siqi Zhang, Qingyuan Yu, Yukun Sun, Guangning Zhang, Yonggen Zhang, Hangshu Xin

**Affiliations:** College of Animal Science and Technology, Northeast Agricultural University, Harbin 150030, China; zhangsiqimax@126.com (S.Z.);

**Keywords:** branched-chain fatty acids, LPS, calf small intestinal epithelial cells, inflammation

## Abstract

This study examined branched-chain fatty acids (BCFAs)’ effects on oxidative stress, energy metabolism, inflammation, tight junction disruption, apoptosis, and Toll-like receptor 4/nuclear factor kappa-B (*TLR4/NF-κB*) signaling in lipopolysaccharide (LPS)-induced calf small intestinal epithelial cells (CSIECs). Eight groups were used: a control group, an LPS-induced group, and six BCFA treatment groups (12-methyltridecanoic acid (iso-C14:0), 13-methyltetradecanoic acid (iso-C15:0), 14-methylpentadecanoic acid (iso-C16:0), 15-methylhexadecanoic acid (iso-C17:0), 12-methyltetradecanoic acid (anteiso-C15:0), and 14-methylhexadecanoic acid (anteiso-C17:0)) with LPS. The BCFA pretreatments significantly increased CSIEC activity compared to the LPS-induced group, with iso-C14:0 showing the highest activity (89.73%). BCFA reduced Reactive Oxygen Species (ROS) generation and malondialdehyde (MDA) levels and improved the superoxide dismutase (SOD), glutathione peroxidase (GSH-Px), and catalase (CAT) activities and glutathione (GSH) levels. Iso-C16:0 optimized total antioxidant capacity (T-AOC). BCFA enhanced the mitochondrial membrane potential, Adenosine Triphosphate (ATP) enzyme activity, and ATP content, with iso-C14:0 increasing ATP by 27.01%. BCFA downregulated interleukin (*IL*)*-1β*, *IL-8*, tumor necrosis factor (*TNF*)-*α*, and interferon (*INF*)-*γ* gene expression, reduced IL-6 levels, and increased *IL-10* expression. Myeloid differentiation factor 88 (*MyD88*) mRNA levels were reduced. BCFA alleviated Zonula Occludin (*ZO-1*), *Claudin-1*, and *Claudin-4* decrease and increased Occludin levels. BCFA mitigated LPS-induced increases in *Caspase-3* and BCL2-Associated X (*BAX*) mRNA levels, reduced *Caspase-8* and *Caspase-9* expression, and increased B-Cell Lymphoma-2 (*BCL-2*) mRNA levels. The Entropy Weight-TOPSIS method was adopted, and it was discovered that iso-C15:0 has the best effect. In summary, BCFA supplementation mitigated oxidative stress and enhanced mitochondrial function. BCFA inhibited *TLR4/NF-κB* signaling pathway overactivation, regulated inflammatory cytokine gene expression, reduced cellular apoptosis, preserved tight junction integrity, and supported barrier function.

## 1. Introduction

Diarrhea is the leading cause of morbidity and mortality in calves, accounting for approximately 56% of calf illnesses and 32% of calf deaths [1]. The condition adversely affects average daily gain, diminishes immune function, and predisposes calves to secondary diseases, resulting in substantial economic losses for the dairy industry [2]. The primary etiological factors of calf diarrhea include inflammation and functional damage to the intestine tract, which may be provoked by various pathogens such as Escherichia coli and Salmonella spp., viral agents like rotavirus and coronavirus, and parasites such as Cryptosporidium and coccidia [3]. Notably, these pathogens have also been detected in the intestines of healthy calves, suggesting that their mere presence may not necessarily correlate with the onset of diarrhea [4]. Clinical interventions often involve the administration of antibiotics, with studies indicating that 74% of calf diarrhea cases are treated with these medications; however, less than half of producers have implemented written treatment protocols developed in consultation with clinical veterinarians [5]. This underscores the necessity for clinical veterinarians to provide an accurate diagnosis of calf diarrhea to mitigate the risks associated with misdiagnosis, which can result in antibiotic overuse and the emergence of antibiotic-resistant bacteria [6]. Furthermore, exposure to antibiotics during the early life of calves can induce alterations in intestinal microflora, leading to gastrointestinal dysfunction and prolonged immunological impairment [7]. In recent years, the beneficial biofunctional effects of branched-chain fatty acids (BCFAs) on gut health have gradually emerged and been confirmed.

BCFAs can be classified into two major categories: monomethyl BCFAs and polymethyl BCFAs. Monomethyl BCFAs predominantly consist of saturated fatty acids, characterized by a branched-chain alkyl group located on the second carbon atom (iso-BCFA) or the third carbon atom (anteiso-BCFA). The presence of BCFAs is prevalent in ruminant-derived products, such as milk, beef, and lanolin, accounting for 2% to 45% of total fatty acids [3,8]. The predominant BCFAs found in ruminants include 12-methyltridecanoic acid (iso-C14:0), 13-methyltetradecanoic acid (iso-C15:0), 12-methyltetradecanoic acid (anteiso-C15:0), 14-methylpentadecanoic acid (iso-C16:0), 15-methylhexadecanoic acid (iso-C17:0), and 14-methylhexadecanoic acid (anteiso-C17:0). Recent studies have highlighted the beneficial role of BCFAs in mitigating intestinal inflammation. For instance, Yan et al. [9] demonstrated that BCFAs significantly enhanced cell viability and reduced the expression of interleukin (*IL*)-8 and nuclear factor kappa-B (*NF-κB*) in lipopolysaccharide (LPS)-stimulated Caco-2 cells while inhibiting Toll-like receptor 4 (*TLR-4*) expression. In vitro studies showed that treatment with 20 mmol/L of BCFAs dose-dependently prevented the decrease in transepithelial electrical resistance (TEER) in Caco-2 cell monolayers induced by tumor necrosis factor (*TNF*)-*α* and interferon (*INF*)-*γ*, indicating a protective effect on intestinal barrier function during inflammatory processes [10]. Moreover, Ran-Ressler et al. [11] found that neonatal Sprague Dawley rats fed a diet enriched with a mixture of BCFAs (20%, wt/wt, including iso-14:0, anteiso-15:0, iso-16:0, anteiso-17:0, iso-18:0, and iso-20:0) exhibited a 56% reduction in the incidence of necrotizing enterocolitis, along with an increased abundance of Bacillus subtilis in the ileum and a threefold elevation in *IL-10* expression. Another recent study identified the presence of BCFAs in calf feces, with their composition and concentration linked to calf intestinal health [3]. These observations suggest that BCFAs could confer benefits to both human and animal health through their potential effects on intestinal function.

The Entropy Weight-TOPSIS method represents a robust multi-attribute decision-making approach. The Entropy Weight Method (EWM) is widely utilized for indicator weighting based on data dispersion, with greater dispersion indicating more significant differences, subsequently allowing for increased weight allocation to more informative indicators [12,13,14]. The Technique for Order Preference by Similarity to Ideal Solution (TOPSIS), first introduced by Hwang and Yoon in 1981 [15], is an effective methodology for addressing multi-attribute decision-making problems involving finite alternatives. This method ranks options based on the computed distances between each alternative and both positive and negative ideal solutions, thereby facilitating the identification of optimal choices [16,17,18,19,20]. EWM is often employed to determine attribute weights for the TOPSIS method [21,22,23]. The influence of each BCFA monomer on different test indicators varies, indicating that the assessment based on a single factor or a main indicator alone has limitations. This algorithm addresses the issue of inconsistent dimensions and measurement values by standardizing the factors. Therefore, the analysis is based solely on all of the data collected during the experiment.

In recent years, the beneficial biofunctional effects of BCFAs on gut health have been substantiated in studies involving humans and rodents [9,11]. However, there remains a paucity of reports and data concerning the notable biological activity of BCFAs within the realm of animal nutrition. In young ruminants, the gastrointestinal system is vital during early development, particularly due to the immature state of their rumen. Consequently, the potential advantages of BCFAs for the gut health development of young ruminants merit in-depth exploration and investigation. Therefore, in this study, we utilized LPS to establish an inflammation model in CSIECs, simulating calf intestinal barrier damage caused by Escherichia coli infection, and aimed to investigate the influence of individual BCFAs (including iso-C14:0, iso-C15:0, iso-C16:0, iso-C17:0, anteiso-C15:0, and anteiso-C17:0) on LPS-induced oxidative stress, energy metabolism, inflammation, tight junction disruption, apoptosis, and the *TLR4/NF-κB* signaling pathway in CSIECs. Subsequently, the Entropy Weight-TOPSIS method was employed to evaluate the activity and effectiveness of BCFA monomers, seeking to elucidate whether BCFA exerts a significant ameliorative effect on LPS-induced inflammatory damage in calf intestinal epithelial cells. This finding will provide novel insights into the potential of BCFAs as nutritional regulatory factors that support the healthy development of the intestine during the early stages of ruminant growth.

## 2. Materials and Methods

### 2.1. Reagents and Materials

The BCFAs (including iso-C14:0, iso-C15:0, iso-C16:0, iso-C17:0, anteiso-C15:0, and anteiso-C17:0) and bovine serum albumin (BSA, fatty acid-free) were obtained from SCR-Biotech (Shanghai, China). The LPS from *E. coli* O55:B5 were purchased from Sigma-Aldrich (Shanghai, China). Dulbecco’s Modified Eagle’s Medium/Ham’s F-12 (DMEM/F12), fetal bovine serum (FBS), and phosphate-buffered saline (PBS) were sourced from SenBeiJia (Nanjing, China). Dimethyl sulfoxide (DMSO) was acquired from Amresco (Washington, WA, USA). A 0.25% trypsin-EDTA solution and penicillin-streptomycin 100X solution were obtained from Beyotime (Shanghai, China). Calf small intestinal epithelial cell lines (CTCC-001-0886) were procured from Zhejiang Meisen Cell Technology (Hangzhou, China).

### 2.2. Cell Culture

The CSIECs were cultured in DMEM/F12 supplemented with 10 ng/mL epidermal growth factor, 5 μg/mL insulin, 10% FBS, and 1% penicillin-streptomycin at 37 °C in a humidified atmosphere containing 5% CO_2_. When the cells reached approximately 80% confluence, they were digested with trypsin and passage.

### 2.3. Establishment of Cell Inflammatory Model and Selection of BCFA Concentration

To determine the optimal concentration of LPS, CSIECs were seeded at a density of 5 × 10^3^ cells/well in 96-well plates, with six replications per treatment. Upon reaching 60–70% confluence, the medium was replaced with FBS-free DMEM/F-12, and the cells were treated with LPS at concentrations of 0, 1, 5, 10, 25, 50, and 100 μg/mL for 24 h to induce inflammation. Similarly, each of the six BCFAs was dissolved in 10% BSA (fatty acid-free) and further diluted in cell culture medium prior to use [24]. To identify the optimal concentration of BCFAs, CSIECs were seeded in 96-well plates at a density of 5 × 10^3^, with six replicates per treatment. After incubation until the cells reached 60–70% confluence, the medium was replaced with FBS-free DMEM/F-12, and various concentrations of BCFAs (0, 0.5, 1, 2.5, 5, 10, and 20 μmol/L) were added. The cells were incubated for an additional 24 h.

Cell viability was assessed using a cell counting kit (CCK-8, EnoGene, Nanjing, China) according to the manufacturer’s instructions. Briefly, 100 μL of FBS-free DMEM/F-12 containing 10 μL of CCK-8 reagent was added to each well. After a 1 h incubation period at 37 °C, absorbance was measured at 450 nm using a microplate reader. Cell viability was calculated using the formula, cell viability = (As − Ab)/(Ac − Ab) × 100%, where “As” denotes the absorbance of the LPS-treated group, “Ac” denotes the absorbance of the untreated group, and “Ab” represents the absorbance of the blank group containing a culture medium and CCK-8 without cells or LPS. The cell viability of the untreated group was considered 100%. Based on the results, a concentration of 10 μg/mL LPS for 24 h, which significantly reduced the cell viability, was selected for subsequent experiments. Due to the high cell viability observed following treatment with 1 μmol/L BCFA, this concentration was selected for further experiments in this study.

### 2.4. Fatty Acid Supplementation and Inflammatory Stimulation

CSIECs were inoculated in culture flasks and allowed to grow until they reached 60~70% confluence. The basal medium was then replaced with FBS-free DMEM/F-12, and the cells were incubated with 1 μmol/L BCFA for 24 h. Following incubation, the medium containing the fatty acid substrates was removed, and the cells were washed twice with PBS. Subsequently, the cells were exposed to LPS at a final concentration of 10 μg/mL for an additional 24 h. After this incubation period, the medium was aspirated, the cells were washed twice with PBS, and they were then digested with trypsin for collection and further experimentation.

### 2.5. Reactive Oxygen Species (ROS) Detection

Detection was performed using a reactive oxygen species detection kit (S0033S ROS, Shanghai Beyotime Biotechnology Co., Shanghai, China). CSIECs were seeded into 6-well plates and cultured in complete medium under 5% CO_2_ at 37 °C. Once the cells reached approximately 80% confluence, they were processed. Then, DCFH-DA was added at a dilution of 1:1000 to achieve a final concentration of 10 μmol/L in serum-free medium. The cells were incubated at 37 °C for 20 min to allow for adequate probe loading. Following incubation, the cells were washed three times with serum-free medium to remove any unincorporated DCFH-DA, and fluorescence was subsequently detected using a fluorescence microscope (APX100, Olympus, Tokyo, Japan).

### 2.6. Detection of Antioxidant Enzymes

When the CSIECs reached 80% confluence and appeared healthy, they were digested with trypsin and transferred to a centrifuge tube. The cells were centrifuged at 1000 rpm for 10 min, and the supernatant was discarded. The cells were then washed with PBS buffer and centrifuged again under the same conditions. Following this, 0.5 mL of PBS buffer was added to the cell pellet, and the cells were lysed using an ultrasonic disintegrator (Q700 Sonicator, Qsonica, Newtown, CT, USA). The content and activity of superoxide dismutase (SOD), catalase (CAT), malondialdehyde (MDA), glutathione (GSH), glutathione peroxidase (GSH-Px), and total antioxidant capacity (T-AOC) were measured using a spectrophotometer or microplate reader. The experimental procedures were conducted according to the instructions provided in the detection kits (A001-1-2 SOD, A007-1-1 CAT, A003-1-2 MDA, A006-2-1 GSH, A005-1-2 GSH-Px, and A015-2-1 T-AOC from Nanjing Jiancheng Bioengineering Institute, Nanjing, China).

### 2.7. Mitochondrial Membrane Potential Assay

The mitochondrial membrane potential was assessed using the mitochondrial membrane potential detection kit (C2006-1 MMP, Shanghai Beyotime Biotechnology Co., Shanghai, China) according to the manufacturer’s instructions. Briefly, when the confluence of CSIECs reached 80%, the culture medium was removed from the 6-well plates. Subsequently, 1 mL of fresh cell culture medium and 1 mL of JC-1 staining working solution (containing green fluorescent J-monomers and red fluorescent J-aggregates) were added and mixed thoroughly. The cells were then incubated at 37 °C for 20 min. After incubation, the cells were washed twice with JC-1 staining buffer (1×), followed by the addition of 2 mL of cell culture medium. Fluorescence was observed using a fluorescence microscope (APX100, Olympus, Tokyo, Japan).

### 2.8. Adenosine Triphosphate (ATP) Enzyme Activity and ATP Content Detection

All kits were sourced from Nanjing Jiancheng Bioengineering Institute (Nanjing, China). When the CSIECs reached 80% confluence, the cell precipitate was collected, and a cell suspension was prepared at a concentration of 10^7^ cell/mL using PBS. The cells were then disrupted using an ultrasonic disintegrator (Q700 Sonicator, Qsonica, Newtown, CT, USA). Following the kit instructions, the activities of four ATPases (Na^+^-K^+^-ATPase, Mg^2+^-ATPase, Ca^2+^-ATPase, and Ca^2+^-Mg^2+^-ATPase; kit number: A016-1-1) were measured using a spectrophotometer (U-2990, HITACHI, Tokyo, Japan).

To assess the ATP content, when the CSIECs reached 80% confluence, the cell precipitate was collected, and 500 μL of cold double-distilled water was added. The mixture was placed in an ice water bath and subjected to ultrasonic disruption. The cell suspension was then heated in a boiling water bath for 10 min, followed by mixing for an additional 1 min to ensure thorough extraction. The ATP content was measured according to the ATP content measurement kit (Kit number: A095-1-1), with absorbance measured at 636 nm. The ATP concentration was calculated according to the formula provided in the instruction manual. Among them, *Ad* represents the absorbance value of the test group; *Ac* represents the absorbance value of the control group; *As* represents the absorbance value of the standard group; *Ab* represents the absorbance value of the blank group; *Cs* represents the concentration of the standard sample, which is 1000 μmol/L; *N* represents the dilution factor before sample measurement; and *Cpr* represents the protein concentration of the homogenate.

$$ATP\;content\;\left(\frac{\mu mol}{gprot}\right)=\frac{Ad-Ac}{As-Ab}*Cs*N÷Cpr$$

### 2.9. RNA Isolation, Reverse Transcription, and Quantitative Real-Time PCR (qRT-PCR)

CSIECs were inoculated in 6-well plates and cultured in FBS-free DMEM/F-12 with 1 μmol/L BCFA for 24 h after reaching 60–70% confluence. Following this incubation period, the FBS-free DMEM/F-12 was replaced with fresh medium, and the cells were treated with LPS at a final concentration of 10 μg/mL for 24 h. At the conclusion of the experiment, the cells were washed twice with ice-cold PBS. Total RNA was extracted using TRIzol reagent (Ambion, Austin, TX, USA) following the manufacturer’s protocol. The content and quality of the total RNA were assessed using a NanoDrop ND-2000 ultra-micro spectrophotometer (DS-11, DENOVIX, Wilmington, DE, USA), ensuring that the OD260/OD280 ratio was between 1.9 and 2.1, meeting the purity requirement for total RNA. The primers used for qRT-PCR are detailed in Table 1, with β-actin serving as an internal reference gene, synthesized by Sangon Biotech Co., Ltd. (Shanghai, China). Reverse transcription of mRNA was performed using a 20 µL reverse transcription reaction system according to the manufacturer’s instructions. qRT-PCR reactions were conducted using a QuantStudio3 real-time PCR instrument (96 wells, 0.2 mL/well, Thermo Fisher Scientific, Waltham, MA, USA). The CT values were analyzed using the 2^−ΔΔCT^ method to determine the relative expression of genes in each group.

### 2.10. Evaluating the Mitigating Effect of BCFAs on LPS-Induced Inflammatory Responses in CSIECs Using the Entropy Weight-TOPSIS Method

#### 2.10.1. Calculation of Entropy Weight-TOPSIS Method

We standardized the original data, calculated the weights of each indicator using the entropy method, and then used the product of the weights and the standardized data as the original data input for the TOPSIS method. We applied the TOPSIS method to compute the proximity degree (the comprehensive evaluation index for each sample) between each evaluated object and the optimal solution. Finally, the evaluation objects were ranked according to the calculated closeness coefficient [21,22,23]. A higher closeness degree indicates a stronger mitigating effect of BCFAs on LPS-induced inflammatory responses in CSIECs.

#### 2.10.2. Selection and Identification of Evaluation Indicators

Based on the various types of detection indicators, four primary indicators were selected for evaluation (refer to Table 2, Table 3, Table 4 and Table 5).

### 2.11. Statistical Analysis

The preliminary organization and calculation of experimental data were conducted using Excel 2019. The one-way ANOVA procedure in SAS 9.4 software was employed to perform significance tests on cell viability, ROS levels, and the expression levels of target gene mRNA. Multiple comparisons were carried out using Duncan’s method, with *p* < 0.05 indicating significant differences. Data analysis and comparison were performed using the Entropy Weight-TOPSIS method. The results obtained from ROS and JC-I fluorescence microscopy detection were analyzed and plotted using ImageJ 1.8.0 software. All other data visualizations were created using GraphPad Prism 9 software.

## 3. Results

### 3.1. Concentration of LPS and BCFAs

**Figure 1:**
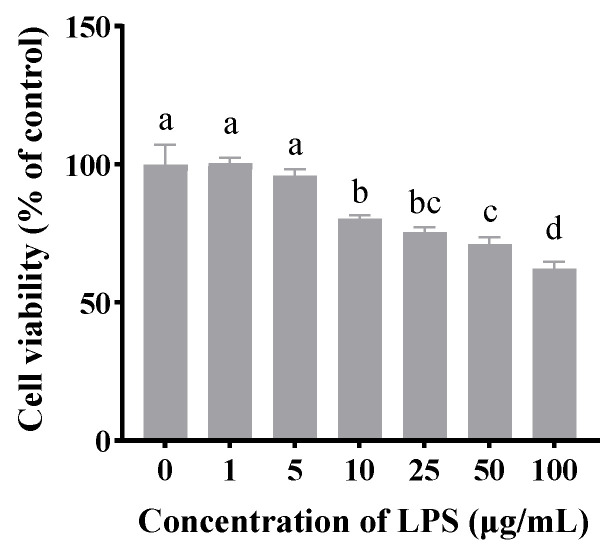
The effects of LPS on the viability of CSIECs using the CCK-8 assay. The cells were treated with various concentrations of LPS (0, 1, 5, 10, 25, 50, and 100 μg/mL) for 24 h. The data are expressed as the mean ± standard deviation (mean ± SD), and significant differences are indicated by different letters (*p* < 0.05).

As shown in Figure 1, compared to the untreated group, LPS concentrations of 10, 25, 50, and 100 μg/mL significantly inhibited cell viability (*p* < 0.05). Notably, treatment with 10 μg/mL LPS for 24 h reduced cell viability to approximately 70%. Therefore, this concentration was selected for subsequent experiments.

**Figure 2:**
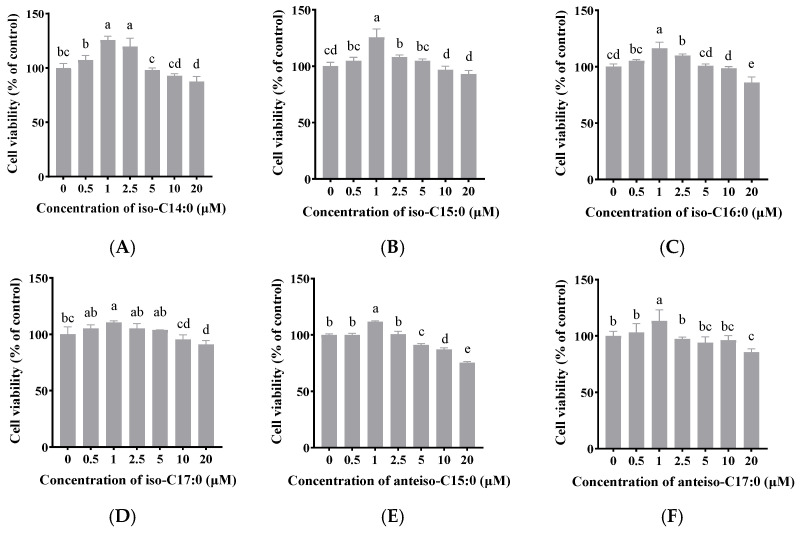
The effects of BCFAs (iso-C14:0 (**A**), iso-C15:0 (**B**), iso-C16:0 (**C**), iso-C17:0 (**D**), anteiso-C15:0 (**E**), and anteiso-C17:0 (**F**)) on the viability of CSIECs determined by the CCK-8 assay. The cells were treated with different concentrations of BCFAs (0, 0.5, 1, 2.5, 5, 10, and 20 μmol/L) for 24 h. The data are expressed as the mean ± standard deviation (mean ± SD), and significant differences are indicated by different letters (*p* < 0.05).

As shown in Figure 2, when the final concentration of BCFAs (iso-C14:0, iso-C15:0, iso-C16:0, iso-C17:0, anteiso-C15:0, and anteiso-C17:0) in FBS-free DMEM/F-12 was 1 μmol/L, cell viability significantly increased to 125.66%, 125.11%, 116.25%, 109.00%, 111.79%, and 119.09%, respectively (*p* < 0.05). Thus, a concentration of 1 μmol/L was selected for the following experiments.

### 3.2. Effect of BCFAs on LPS-Induced Viability of CSIECs

**Figure 3:**
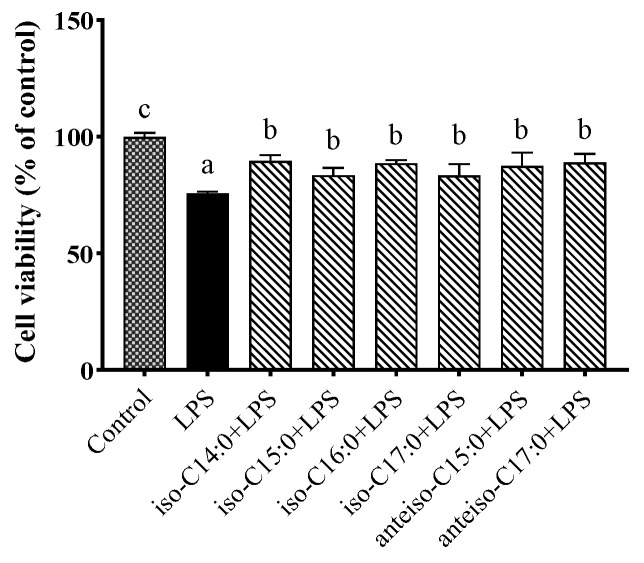
The effects of BCFAs on the viability of CSIECs induced by LPS. The cells were pretreated with BCFAs (iso-C14:0, iso-C15:0, iso-C16:0, iso-C17:0, anteiso-C15:0, and anteiso-C17:0; 1 μmol/L) for 24, followed by LPS (10 μg/mL) treatment for another 24 h. The data are expressed as the mean ± standard deviation (mean ± SD), and significant differences are indicated by different letters (*p* < 0.05).

Figure 3 demonstrates that the stimulation of CSIECs with 10 µg/mL of LPS for 24 h significantly reduced cell viability to approximately 75.78% (*p* < 0.05). However, pretreatment with 1 µmol/L of BCFAs significantly enhanced cell viability (*p* < 0.05). Among the various kinds of BCFAs, iso-C14:0 pretreatment resulted in the highest cell viability at 89.73% (*p* < 0.05), followed by anteiso-C17:0 (89.11%), iso-C16:0 (88.65%), anteiso-C15:0 (87.52%), iso-C15:0 (83.61%), and iso-C17:0 (83.40%). No significant differences were observed among the viability results of the different BCFA treatments (*p* > 0.05).

### 3.3. Effect of BCFAs on ROS Level in LPS-Induced CSIECs

**Figure 4:**
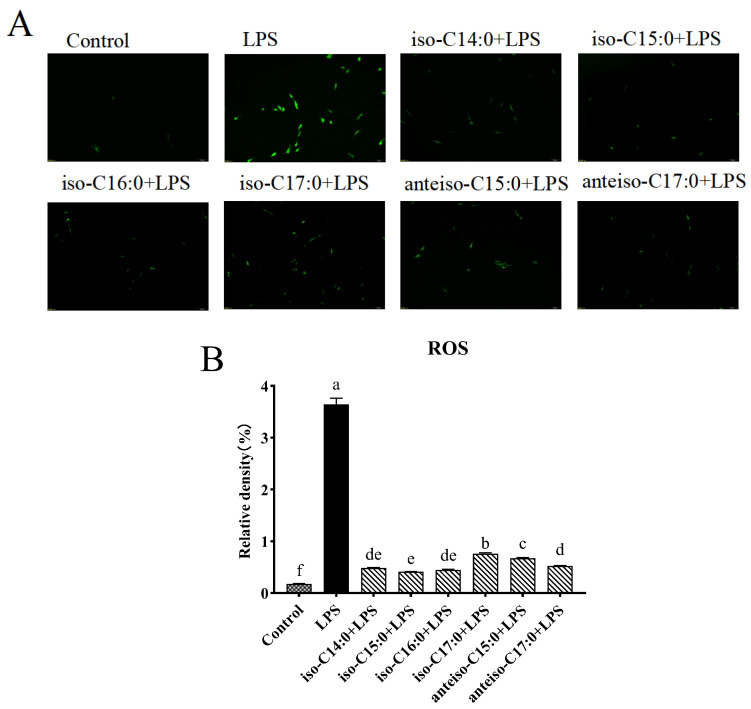
Effects of BCFA on ROS in CSIEC induced by LPS. The green fluorescence indicates the level of ROS within the cells. (**A**) Fluorescence microscopic observation of reactive oxygen staining (scale bar = 100 μm). (**B**) Fluorescence density value analysis of fluorescent pictures. Data are expressed as mean ± standard deviation (mean ± SD), and significant differences are indicated by different letters (*p* < 0.05).

As illustrated in Figure 4, LPS treatment significantly elevated ROS levels in CSIECs, increasing to 20.80 times that of the control (*p* < 0.05). In contrast, treatment with BCFAs resulted in a marked reduction in ROS production, as indicated by decreased fluorescence intensity (*p* < 0.05) The efficacy of BCFAs in inhibiting ROS production, ranked by effectiveness, was as follows: iso-C15:0, iso-C16:0, iso-C14:0, anteiso-C17:0, anteiso-C15:0, and iso-C17:0 (*p* < 0.05).

### 3.4. Effect of BCFAs on Oxidative Stress Indicators in LPS-Induced CSIECs

**Figure 5:**
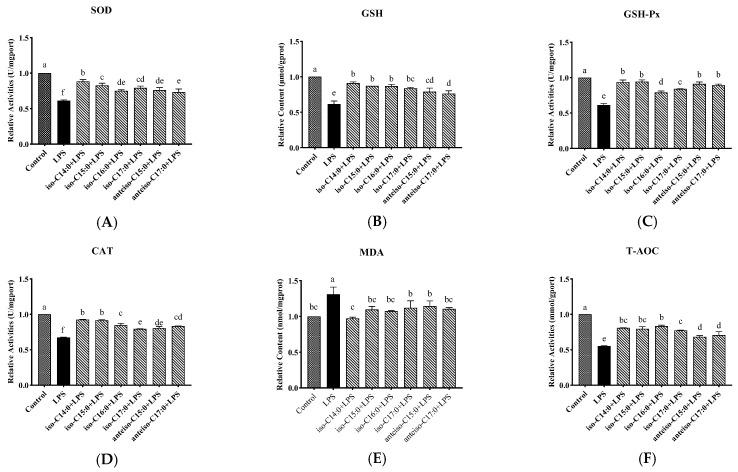
Effects of BCFAs on oxidative stress indicators in CSIECs induced by LPS. (**A**) SOD activity, (**B**) GSH content, (**C**) GSH-Px activity, (**D**) CAT activity, (**E**) MDA content, and (**F**) T-AOC activity. Data are expressed as mean ± standard deviation (mean ± SD), and significant differences are indicated by different letters (*p* < 0.05).

The results shown in Figure 5 indicate that, compared to the control, LPS treatments significantly decreased SOD, GSH-Px, CAT, and T-AOC activities, as well as the GSH content (*p* < 0.05), while the MDA content significantly increased (*p* < 0.05). However, pretreatment with BCFAs prior to LPS stimulation improved the oxidative stress profile. Iso-C14:0 showed the most significant enhancement in SOD activities. In contrast, iso-C16:0 demonstrated the highest relative T-AOC activity. These findings suggest that BCFAs can alleviate oxidative stress in CSIECs induced by LPS.

### 3.5. Effect of BCFAs on Mitochondrial Membrane Potential in LPS-Induced CSIECs

**Figure 6:**
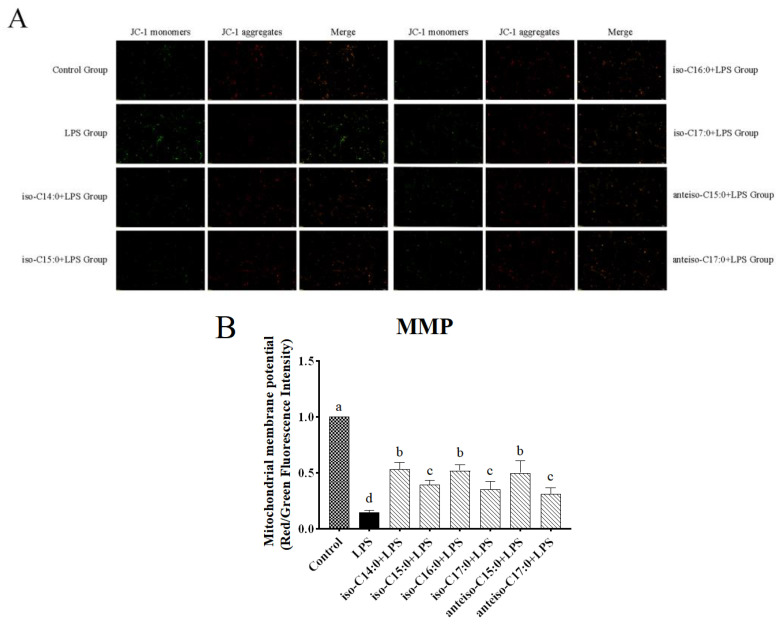
Effects of BCFAs on MMP in CSIECs induced by LPS. The intensity of red fluorescence reflects a state where the mitochondrial membrane potential is at a relatively high level, while the emergence of green fluorescence indicates a decrease in the mitochondrial membrane potential. (**A**) Fluorescence microscopic observation of reactive oxygen staining (scale bar = 100 μm). (**B**) Fluorescence density value analysis of fluorescent pictures. Data are expressed as mean ± standard deviation (mean ± SD), and significant differences are indicated by different letters (*p* < 0.05).

According to Figure 6, in the control, JC-1 exists in a polymeric form in the mitochondria of normal CSIECs, exhibiting bright red fluorescence, while the green fluorescence in the cells is very weak, suggesting vigorous cell metabolism. Reducing MMP could monotonize JC-1 with a switch from red to green fluorescence. After LPS stimulation, the red fluorescence intensity in the mitochondria decreases significantly, and the green fluorescence in the cytoplasm increases significantly. The fluorescence microscope observation results show that when CSIECs were pretreated with BCFAs before LPS stimulation, the green fluorescence decreased and the red fluorescence increased. Compared to the LPS group, the red/green fluorescence intensity ratios of mitochondrial membrane potential in the various BCFA groups increased by 38.55%, 24.83%, 37.30%, 20.60%, 35.23%, and 16.29%, respectively, all showing significant increases (*p* < 0.05).

### 3.6. Effect of BCFAs on ATPase Activity in LPS-Induced CSIECs

**Figure 7:**
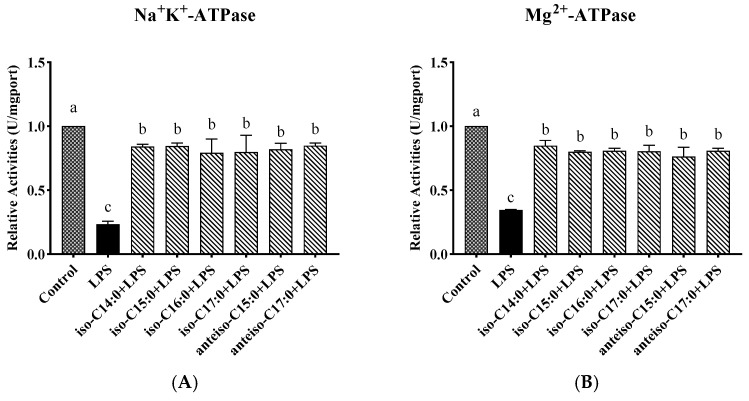

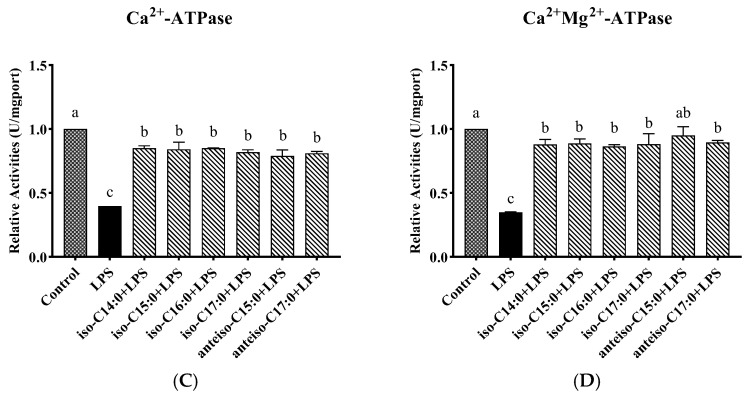
Effects of BCFAs on ATPase activity of CSIECs induced by LPS. (**A**) Na^+^-K^+^-ATPase activity, (**B**) Mg^2+^-ATPase activity, (**C**) Ca^2+^-ATPase activity, and (**D**) Ca^2+^-Mg^2+^-ATPase activity. Data are expressed as mean ± standard deviation (mean ± SD), and significant differences are indicated by different letters (*p* < 0.05).

According to Figure 7, compared to the control, the activities of Na^+^-K^+^-ATPase, Mg^2+^-ATPase, Ca^2+^-ATPase, and Ca^2+^-Mg^2+^-ATPase markedly decreased by 23.50%, 34.59%, 39.81%, and 34.88%, respectively (*p* < 0.05). In contrast, the BCFA pretreatment yielded significant increases in ATPase activities compared to the LPS group (*p* < 0.05). Notably, the activity of Ca^2+^-Mg^2+^-ATPase in the anteiso-C15:0 group showed a significant increase and did not differ from the control (*p* > 0.05).

### 3.7. Effect of BCFAs on ATP Content in LPS-Induced CSIECs

**Figure 8:**
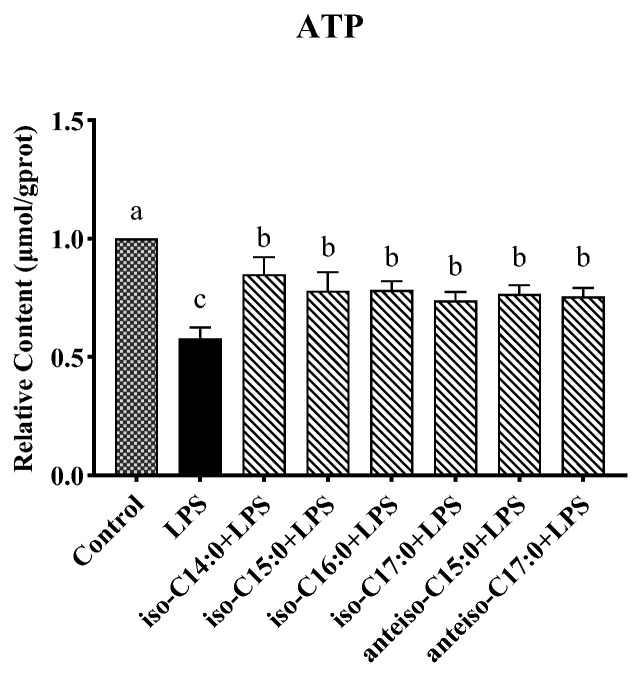
Effects of BCFAs on ATP content in CSIECs induced by LPS. Data are expressed as mean ± standard deviation (mean ± SD), and significant differences are indicated by different letters (*p* < 0.05).

As seen from Figure 8, LPS stimulation resulted in a significant decrease in the ATP content (*p* < 0.05). The ATP levels remained significantly lower in the BCFA groups compared to the control (*p* < 0.05), and they were significantly higher than in the LPS group (*p* < 0.05). Among the BCFA treatments, the iso-C14:0 group exhibited the highest ATP content, with a 27.01% increase compared to the LPS group. The ATP contents in the remaining BCFA groups, ranked from highest to lowest, were iso-C16:0, iso-C15:0, anteiso-C15:0, anteiso-C17:0, and iso-C17:0.

### 3.8. Effects of BCFAs on Expression Levels of Cytokine mRNA in LPS-Induced CSIECs

**Figure 9:**
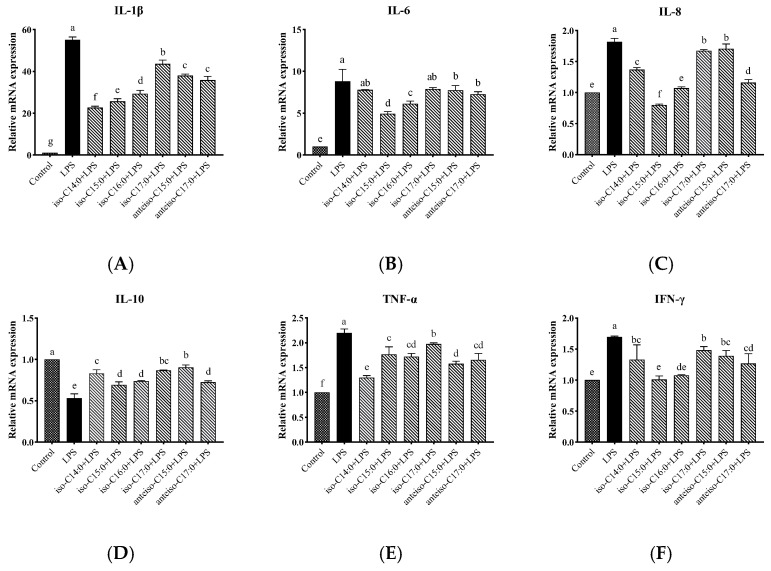
Effects of BCFAs on cytokine mRNA expression levels in CSIECs induced by LPS. (**A**) *IL-1β* gene expression, (**B**) *IL-6* gene expression, (**C**) *IL-8* gene expression, (**D**) *IL-10* gene expression, (**E**) *TNF-α* gene expression, and (**F**) *IFN-γ* gene expression. Data are expressed as mean ± standard deviation (mean ± SD), and significant differences are indicated by different letters (*p* < 0.05).

As shown in Figure 9, compared to the control, LPS stimulation significantly increased the relative mRNA expression levels of *IL-1β*, *IL-6*, *IL-8*, *TNF-α*, and *IFN-γ* (*p* < 0.05). Additionally, the expression level of *IL-10* has been significantly reduced (*p* < 0.05) (Figure 9D). In contrast, pretreatment with BCFAs significantly reduced the mRNA expression levels of *IL-1β*, *IL-8*, *TNF-α*, and *IFN-γ* (*p* < 0.05).

Specifically, iso-C14:0 pretreatment showed the most significant reduction in *IL-1β* expression (*p* < 0.05), followed by iso-C15:0, iso-C16:0, anteiso-C17:0, anteiso-C15:0, and iso-C17:0 (*p* < 0.05). For *IL-6*, iso-C15:0, iso-C16:0, anteiso-C15:0, and anteiso-C17:0 exhibited significantly lower expression compared to the LPS group (*p* < 0.05), with iso-C15:0 yielding the lowest values. No significant differences were observed in the *IL-6* mRNA levels between the LPS group and the iso-C14:0 or iso-C17:0 treatments (*p* > 0.05). All BCFA treatments showed significant protective effects on *IL-8* expression (*p* < 0.05), with efficacy ranked as follows: iso-C15:0, anteiso-C17:0, iso-C16:0, iso-C14:0, iso-C17:0, and anteiso-C15:0 (*p* < 0.05).

Treatments with BCFAs also significantly enhanced the relative mRNA expression of *IL-10* (*p* < 0.05), with anteiso-C15:0 showing the greatest improvement, followed by iso-C17:0, iso-C14:0, iso-C16:0, anteiso-C17:0, and iso-C15:0 (*p* < 0.05). For *TNF-α*, the downregulation effects of BCFAs were ranked from highest to lowest as follows: iso-C17:0, iso-C15:0, iso-C16:0, anteiso-C17:0, anteiso-C15:0, and iso-C14:0 (*p* < 0.05). Additionally, iso-C15:0 and iso-C16:0 exhibited the most effective pretreatment effects on *IFN-γ* expression (*p* < 0.05), followed by anteiso-C17:0, iso-C14:0, anteiso-C15:0, and iso-C17:0 (*p* < 0.05).

### 3.9. Effects of BCFAs on mRNA Expression Levels of Genes Related to TLR4/NF-κB Pathway in LPS-Induced CSIECs

**Figure 10:**
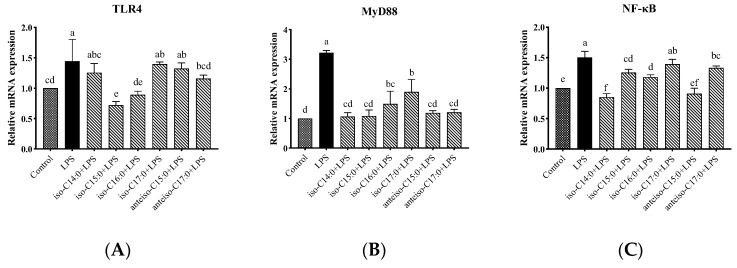
Effects of BCFAs on mRNA expression levels of *TLR4/NF-κB* pathway-related genes in CSIECs induced by LPS. (**A**) *TLR4* gene expression, (**B**) Myeloid differentiation factor 88 (*MyD88*) gene expression, and (**C**) *NF-κB* gene expression. Data are expressed as mean ± standard deviation (mean ± SD), and significant differences are indicated by different letters (*p* < 0.05).

As shown in Figure 10, compared to the control, LPS stimulation significantly increased the relative mRNA expression levels of *TLR4*, Myeloid differentiation factor 88 (*MyD88*), and *NF-κB* in the cells (*p* < 0.05). In cells pretreated with BCFAs, the relative mRNA expression level of *MyD88* significantly decreased compared to the LPS group (*p* < 0.05), with the most pronounced effects being observed in the iso-C14:0, iso-C15:0, anteiso-C15:0, and anteiso-C17:0 groups, followed by iso-C16:0 and iso-C17:0 (*p* < 0.05). Additionally, iso-C15:0, iso-C16:0, and anteiso-C17:0 significantly reduced the relative mRNA expression levels of *TLR4* (*p* < 0.05). Pretreatment with iso-C14:0, iso-C15:0, iso-C16:0, and anteiso-C15:0 significantly downregulated the relative mRNA expression of *NF-κB* (*p* < 0.05). Other BCFA treatments did not significantly affect *TLR4* and *NF-κB* expression levels compared to the LPS group, although a decreasing trend was noted (*p* > 0.05).

### 3.10. Effects of BCFAs on mRNA Expression Levels of Tight Junction Protein-Related Genes in LPS-Induced CSIECs

**Figure 11:**
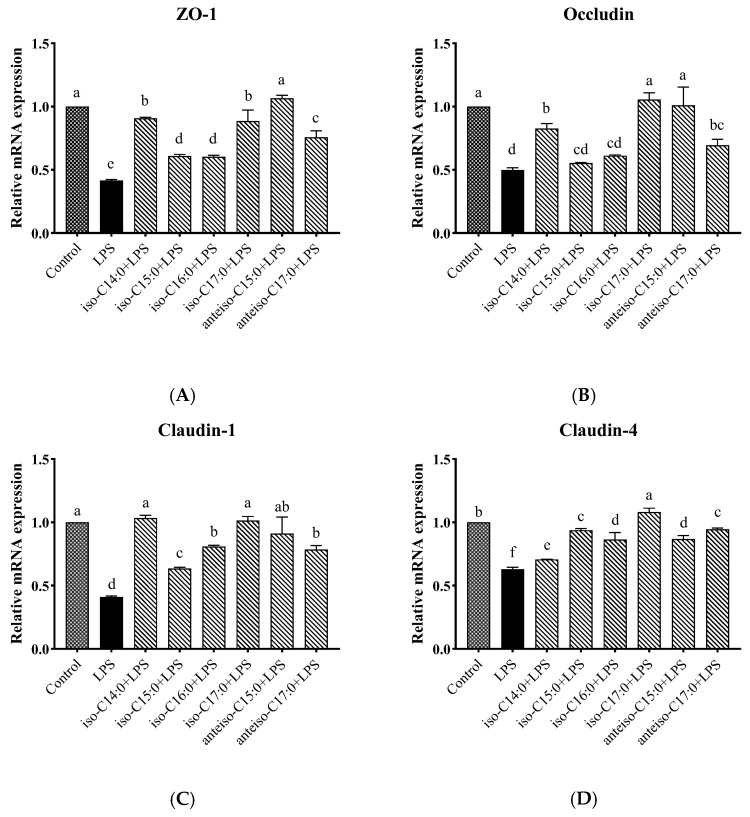
Effects of BCFAs on mRNA expression level of tight junction protein-related genes in CSIECs induced by LPS. (**A**) Zonula Occludin (*ZO-1*) gene expression, (**B**) *Occludin* gene expression, (**C**) *Claudin-1* gene expression, and (**D**) *Claudin-4* gene expression. Data are expressed as mean ± standard deviation (mean ± SD), and significant differences are indicated by different letters (*p* < 0.05).

As shown in Figure 11, LPS stimulation significantly decreased the relative mRNA expression levels of *ZO-1*, *Occludin*, *Claudin-1*, and *Claudin-4* compared to the control (*p* < 0.05). However, pretreatment with BCFA prior to LPS stimulation restored the mRNA expression levels of *ZO-1*, *Occludin*, *Claudin-1*, and *Claudin-4* to varying degrees (*p* < 0.05).

Cells treated with anteiso-C15:0 exhibited the highest mRNA expression level of *ZO-1* (*p* < 0.05), followed by iso-C14:0, iso-C17:0, anteiso-C17:0, iso-C15:0, and iso-C16:0, all significantly higher than the LPS group (*p* < 0.05). For *Occludin* expression, BCFA pretreatment with iso-C14:0, iso-C17:0, anteiso-C15:0, and anteiso-C17:0 resulted in significant increases compared to the LPS group (*p* < 0.05). Among these, iso-C17:0 and anteiso-C15:0 showed the most effective results, with no significant difference from the control (*p* > 0.05). The *Occludin* expression levels in the iso-C15:0 and iso-C16:0 groups did not differ significantly from those in the LPS group (*p* > 0.05), though a trend of increase was observed. With respect to *Claudin-1*, iso-C14:0 and iso-C17:0 demonstrated the best pretreatment effects (*p* < 0.05), with expression levels not significantly different from the control (*p* > 0.05), but significantly higher than those observed in other BCFA groups and the LPS-stimulated group (*p* < 0.05). The relative effects of other BCFAs on *Claudin-1* gene expression in CSIECs, in descending order, were anteiso-C15:0, iso-C16:0, anteiso-C17:0, and iso-C15:0 (*p* < 0.05). The relative mRNA expression results of *Claudin-4* showed that the expression level in the iso-C17:0+LPS group was significantly higher than that in all other groups, including the control (*p* < 0.05). The pretreatment effects of other BCFAs, in descending order, were anteiso-C17:0, iso-C15:0, anteiso-C15:0, iso-C16:0, and iso-C14:0 (*p* < 0.05).

### 3.11. Effects of BCFAs on mRNA Expression Levels of Caspase-Related Genes in LPS-Induced CSIECs

**Figure 12:**
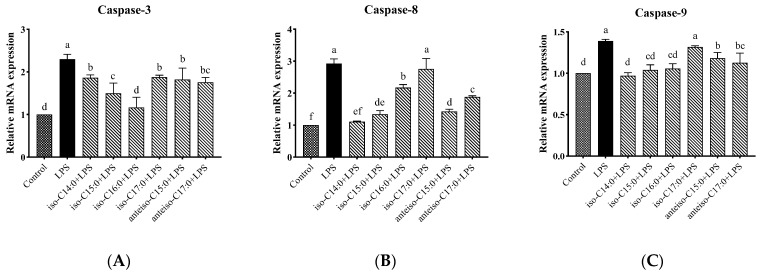
Effects of BCFAs on mRNA expression level of Caspase-related genes in CSIECs induced by LPS. (**A**) *Caspase-3* gene expression, (**B**) *Caspase-8* gene expression, and (**C**) *Caspase-9* gene expression. Data are expressed as mean ± standard deviation (mean ± SD), and significant differences are indicated by different letters (*p* < 0.05).

As shown in Figure 12, compared to the control, the expression levels of *Caspase-3*, *Caspase-8*, and *Caspase-9* genes were significantly increased in the LPS group (*p* < 0.05). In contrast, the BCFA pretreatment significantly reduced the relative expression level of *Caspase-3* in CSIECs compared to LPS stimulation alone (*p* < 0.05). Among these BCFAs, iso-C16:0 showed the lowest expression level, showing no significant difference from the control (*p* > 0.05). The remaining BCFA pretreatment effects, in descending order, were iso-C15:0, anteiso-C17:0, anteiso-C15:0, iso-C14:0, and iso-C17:0 (*p* < 0.05). For the *Caspase-8* gene, all BCFA pretreatments, except for iso-C17:0, significantly reduced the gene expression levels in CSIECs (*p* < 0.05). The effectiveness of the pretreatments was ranked as follows: iso-C14:0, iso-C15:0, anteiso-C15:0, anteiso-C17:0, and iso-C16:0 (*p* < 0.05). Additionally, the relative expression level of the *Caspase-8* gene in the iso-C14:0+LPS group was comparable to that of the control (*p* > 0.05). Similarly, all BCFA pretreatments, except for iso-C17:0, significantly reduced the relative gene expression levels of *Caspase-9* (*p* < 0.05). The expression levels following treatment with iso-C14:0, iso-C15:0, and iso-C16:0 did not differ significantly from the control (*p* > 0.05).

### 3.12. Effects of BCFA on mRNA Expression Levels of BCL2-Associated X (BAX) and B-Cell Lymphoma-2 (BCL-2) Genes in LPS-Induced Calf Small Intestine Epithelial Cells

**Figure 13:**
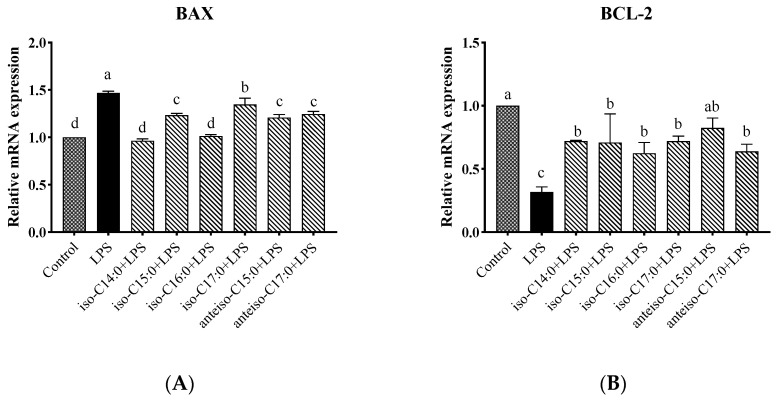
Effects of BCFAs on mRNA expression levels of *BAX* and *BCL-2* genes in CSIECs induced by LPS. (**A**) *BAX* gene expression; (**B**) *BCL-2* gene expression. Data are expressed as mean ± standard deviation (mean ± SD), and significant differences are indicated by different letters (*p* < 0.05).

As shown in Figure 13, compared to the control, the LPS group showed a significant increase in the relative expression level of the *BAX* gene (*p* < 0.05). Following BCFA pretreatment, the relative expression level of the *BAX* gene was significantly reduced compared to the LPS group (*p* < 0.05). Among the treatments, the lowest *BAX* gene expression levels were observed in the iso-C14:0 and iso-C16:0 groups, which did not differ significantly from the control (*p* > 0.05). The protective efficacy of the other BCFA treatments, in descending order, was anteiso-C15:0, iso-C15:0, anteiso-C17:0, and iso-C17:0. Conversely, the relative expression trend of the *BCL-2* gene was opposite to that of the *BAX* gene. Anteiso-C15:0 demonstrated the greatest protective effect, with the following BCFA treatments ranking in descending order of their protective effects: anteiso-C15:0, iso-C17:0, iso-C14:0, iso-C15:0, anteiso-C17:0, and iso-C16:0.

### 3.13. Evaluating the Mitigating Effects of BCFAs on LPS-Induced Inflammatory Responses in CSIECs Using the Entropy Weight-TOPSIS Method

All detected indicator data were standardized, yielding the results presented in Table 6. Then, using the standardized results, a weighted matrix was constructed. Entropy values, weights, and positive and negative ideal solutions were calculated based on a predefined formula. The detailed results are shown in Table 7.

The analysis of these data provides essential support for subsequent processing and analysis, enhancing the authenticity and reliability of the research findings.

To ensure a comprehensive and accurate analysis of the effects of BCFAs on CSIECs, the closeness values and ranking orders of overall indicators, as well as specific indicator categories such as oxidative stress-related indicators, energy metabolism-related indicators, anti-inflammatory property-related indicators, and apoptosis-related indicators were calculated (Table 8).

The ranking order of closeness values for overall indicators is as follows: iso-C15:0 > iso-C14:0 > iso-C16:0 > anteiso-C15:0 > anteiso-C17:0 > iso-C17:0.

The ranking order of closeness values for oxidative stress-related indicators is as follows: iso-C14:0 > iso-C15:0 > iso-C16:0 > iso-C17:0 > anteiso-C17:0 > anteiso-C15:0.

The ranking order of closeness values for energy metabolism-related indicators is as follows: iso-C14:0 > anteiso-C15:0 > iso-C15:0 > iso-C16:0 > anteiso-C17:0 > iso-C17:0.

The ranking order of closeness values for anti-inflammatory property-related indicators is as follows: iso-C15:0 > iso-C14:0 > iso-C16:0 > anteiso-C15:0 > iso-C17:0 > anteiso-C17:0.

The ranking order of closeness values for apoptosis-related indicators is as follows: iso-C16:0 > iso-C15:0 > iso-C14:0 > anteiso-C15:0 > anteiso-C17:0 > iso-C17:0.

## 4. Discussion

Cell viability is a crucial indicator for assessing cell growth, proliferation, and metabolic status [25]. When the body is exposed to external adverse stimuli, it triggers an inflammatory response, which is a dynamic balance process involving the coordination and balance between pro-inflammatory and anti-inflammatory mechanisms. Currently, there are no reports on the establishment of an inflammation model specifically for CSIEC. Zhao [26] and Shi [25] treated IPEC-J2 cells with 10 μg/mL of LPS for 24 h, resulting in changes in tight junctions and impaired cell integrity. In contrast, the addition of BCFAs significantly preserved the viability of LPS-induced CSIECs, demonstrating a clear protective effect. This finding aligns with the results of Yan et al. [9], who treated Caco-2 cells with seven BCFA monomers (including iso-C14:0, iso-C16:0, iso-C18:0, iso-C20:0, anteiso-C13:0, anteiso-C15:0, and anteiso-C17:0) at a concentration of 25 μmol/L each for 24 h. They found that these BCFA monomers effectively mitigated the reduction in cell viability induced by LPS. Notably, BCFAs with shorter carbon chain lengths, such as iso-C14:0, iso-C16:0, and anteiso-C13:0, exhibited a greater ability to maintain cell viability.

Excessive production of ROS or a decrease in antioxidant enzyme activity can overwhelm the antioxidant system, preventing it from neutralizing ROS in a timely manner and leading to oxidative stress as the oxidative processes dominate [27]. Consequently, cellular oxidative stress is closely related to the production and clearance of ROS. A recent study found that stimulating bovine mammary epithelial cells with LPS increased the fluorescence intensity of stromal interaction molecule 1 (STIM1) and orai calcium release-activated calcium modulator 1 (ORAI1) proteins, promoted the nuclear translocation of p65, enhanced mitochondrial ROS production, and elevated mitochondrial membrane potential [28]. Similarly, Li [29] reported that LPS stimulation of bovine mammary epithelial cells resulted in increased ROS release; disrupted the balance of the cellular antioxidant system; led to the oxidation of lipids, proteins, and DNA; and caused damage to surrounding tissues. Our findings these results, showing that LPS stimulation of CSIECs could increase ROS levels. In our study, each BCFA monomer effectively reduced ROS production in LPS-stimulated CSIECs, indicating that BCFAs can decrease peroxidation during the metabolic processes of inflammatory cells. This reduction in ROS levels helps protect cells from free radical damage and mitigates the adverse effects of external factors. While studies on the impact of BCFAs on ROS levels in the body are limited, Mika et al. [30] found that serum levels of C-reactive protein (CRP) and insulin were significantly and negatively correlated with levels of iso-BCFA (iso-C15:0, iso-C16:0, iso-C17:0, anteiso-C15:0, and anteiso-C17:0) in individuals with obesity compared to those without obesity. Given that obesity is often associated with inflammation in body tissues, we can speculate that iso-BCFA may exert beneficial anti-inflammatory effects on the body.

Oxidative stress is an immune response triggered by pathogenic microorganisms and results from the excessive production of ROS [31]. In cells, the extent of oxidative stress can be quantified by measuring MDA levels [32]. Mammalian cells possess antioxidant defense mechanisms that can counteract oxidative stress, employing enzymes such as GSH-Px, CAT, and SOD to mitigate peroxidation reactions [33,34,35]. Our data echo these findings, suggesting that when toxic substances enter CSIECs, they stimulate the production of lipid peroxides such as MDA, increasing peroxidation and cellular damage. Treatment with BCFAs showed positive effects on the cellular antioxidant defense system. In field trials, calves with diarrhea exhibited significantly higher levels of ROS and MDA in their serum and feces compared to healthy calves, indicating ongoing damage to intestinal cells [36]. Conversely, serum CAT activity was lower in the diarrheal calves, but as the diarrhea resolved, oxidative stress indicators in the serum and feces showed improvement [36]. These indicators change with the animal’s physiological state and are considered markers of oxidative stress in the body [36]. These biomarkers fluctuate with the animal’s physiological state and are recognized as indicators of oxidative stress in the body [36]. The results of our experiments align with these principles, suggesting that the addition of BCFAs may help repair damage to CSIECs under stress conditions.

Mitochondrial membrane potential is a crucial indicator of mitochondrial oxidative phosphorylation and overall mitochondrial function, reflecting the cellular redox state. A stable mitochondrial membrane potential is essential for ATP production and serves as a marker of mitochondrial integrity [37]. The ATPase located on the mitochondrial membrane maintains the electrochemical gradient across the inner mitochondrial membrane, and any mitochondrial damage can impair ATPase synthesis [38]. ATPase catalyzes the hydrolysis of ATP, releasing energy necessary for various biological reactions and playing a vital role in cellular energy conversion and utilization [39]. A decrease in mitochondrial membrane potential is a hallmark event in the early stages of apoptosis [40]. Studies have shown that deoxynivalenol disrupts the barrier function of IPEC-J2 cells by inducing the opening of mitochondrial permeability transition pores, thus reducing mitochondrial membrane potential and altering the expression of mitochondrial fission, fusions, and autophagy factors, leading to mitochondrial dysfunction [41]. In our experiments, LPS induction indeed reduced the mitochondrial membrane potential of CSIEC and inhibited the activities of Na^+^-K^+^-ATPase, Mg^2+^-ATPase, Ca^2+^-ATPase, and Ca^2+^-Mg^2+^-ATPase, which interfered with the cell’s mitochondrial energy release and indicated mitochondrial dysfunction induced by LPS. One early specific manifestation of apoptosis is ROS-induced mitochondrial damage, which leads to the loss of mitochondrial membrane potential [42]. The inhibition of ATPase activity can result in mitochondrial Ca^2+^ overload, further compromising mitochondria structure and function, disrupting ion transmembrane transport, and impairing cellular membrane function [43]. Pre-treatment with BCFAs significantly restored mitochondrial membrane potential, increased ATPase activity, elevated ATP levels, alleviated mitochondrial damage, enhanced mitochondrial function, and reduced inflammatory responses. While there is currently no study directly comparing the effects of BCFAs on cellular energy metabolism and mitochondrial function with our findings, a recent review by Yehia et al. [44] conducted in 2023 summarized some associations between BCFAs and cardiovascular diseases. Notably, elevated blood levels of BCFAs may corelate negatively with biomarkers of obesity, insulin sensitivity, and inflammation, suggesting a positive effect of BCFAs on energy metabolism in the body. Nevertheless, the specific targets and underlying mechanisms warrant further investigation.

Excessive inflammatory cytokines can have detrimental effects on the body. Key inflammatory cytokines, including *IL-1β*, *IL-8*, *IL-10*, and *TNF-α*, serve as crucial mediators in the initiation and progression of inflammation, influencing both inflammatory responses and disease development [45]. In the study by Meng et al. [28], the mRNA expression levels of *IL-1β*, *IL-8*, and TNF-α significantly increased in bovine mammary epithelial cells treated with LPS, an observation that was supported by similar results from Tsugami et al. [46]. Numerous studies indicated that substances with antioxidant properties can effectively mitigate oxidative damage caused by LPS. For instance, baicalin can reduce LPS-induced oxidative stress in IPEC-J2 cells, decreasing the expression of *IL-1β*, *IL-6*, *TLR4*, and *TNF-α* genes in these cells [47]. Additionally, koumine has been shown to alleviate elevated levels of *IL-1β* and *IL-6* in LPS-induced IPEC-J2 cells [48]. BCFAs have demonstrated notable anti-inflammatory effects in several studies. For example, Yan et al. [9] reported that application of seven BCFA monomers to LPS-induced Caco-2 cells led to the reduced gene expression of *IL-8*, *NF-κB*, and *TLR4*. Similarly, Ran-Ressler et al. [11] found that feeding newborn rats a diet containing a mixture of BCFAs resulted in a three-fold increase in the expression of the ileal *IL-10* gene. Collectively, these findings imply that BCFAs possess antioxidant properties that can effectively alleviate the oxidative damage inflicted by LPS, thereby minimizing their toxic effects. Consequently, in examinations of inflammatory cytokines related to LPS-stimulated CSIECs, BCFAs were shown to reduce the relative expression levels of pro-inflammatory cytokine genes, mitigating inflammatory damage.

*TLR4* is a transmembrane receptor capable of recognizing and responding to various pathogen-associated molecular patterns (PAMPs) and damage-associated molecular patterns (DAMPs) [49]. Upon *TLR4* activation, *MyD88* interacts with its intracellular domain, acting as a bridge for signal transduction that connects downstream signaling molecules to this pathway [50]. The activation of *MyD88* subsequently triggers pathways such as *NF-κB*, which regulate the production and release of various inflammatory mediators [51]. Importantly, *TLR4* expression is not confined to immune cells; it is found in a variety of cell lines as well [52,53]. For instance, Lactobacillus plantarum 17-5 alleviates Escherichia coli-induced inflammatory responses in bovine mammary epithelial cells by inhibiting *NF-κB* pathway activation. This is evidenced by reduced cell apoptosis rates and downregulated mRNA expression levels of *TLR2*, *TLR4*, *MyD88*, *IL-1β*, *IL-6*, *IL-8*, and *TNF-α*, along with the inhibition of *NF-κB* signaling pathway activation through the suppression of *p65* and *IκBα* phosphorylation [54]. In this study, BCFAs were found to inhibit the expression of *TLR4* and *MyD88* genes while significantly reducing *NF-κB* gene expression in CSIECs, effectively suppressing the inflammatory response. Likewise, Yan [9] found that certain BCFA monomers (e.g., anteis-C13:0 and anteiso-C15:0) inhibited *TLR4* gene expression while significantly reducing *IL-8* and *NF-κB* gene expression in LPS-stimulated Caco-2 cells. Among the fatty acids studied, C12:0 and C16:0, which exhibit similar anti-inflammatory activities, have also been shown to activate the *TLR4* gene on the cell surface [55]. However, data from this experiment indicate that the six BCFA monomeric fatty acids had varying effects on the expression levels of genes associated with the *NF-κB* pathway, suggesting that multiple pathways may be involved in alleviating intracellular inflammatory responses.

Previous studies have demonstrated that intestinal inflammation can compromise the tight junctions between small intestinal epithelial cells, increasing their permeability and disrupting their barrier function [56,57]. For instance, when IPEC-J2 cells are subjected to inflammatory stimulation, the expression levels of tight junction proteins (*ZO-1*, *Occludin*, *Claudin-1*, or *Claudin-4*) will decrease, thereby disrupting the intestinal epithelial barrier function [58]. Furthermore, the incidence of calf diarrhea is correlated with the expression of the *Claudin-1* gene in the jejunum [59]. Our research indicates that LPS stimulation reduces the expression of tight junction protein genes in CSIECs. However, supplementation with BCFAs ameliorates the inhibitory effect of LPS on these proteins. This finding suggests that LPS has a significant toxic impact on the intestine, disrupting its barrier function, while BCFA supplementation appears to mitigate this toxicity. Improving intestinal epithelial barrier function may be one mechanism through which BCFAs exert their anti-inflammatory effects.

Apoptosis, a programmed cell death process, is systematically regulated by genes [60,61,62]. Zhao et al. [26] found that LPS exposure increased levels of apoptotic genes (*Caspase-3*, *Caspase-8*, *Caspase-9*, and *BAX*) and elevated the apoptosis rate in IPEC-J2 cells. Our experiment yielded similar results. However, pretreatment with BCFAs significantly reduced the expression of *Caspase-3*, *Caspase-8*, *Caspase-9*, and *BAX* while increasing *BCL-2* levels. These results indicate that LPS activates the expression of Caspase family-related genes, promoting cell apoptosis in CSIECs. BCFAs exhibited a protective effect against this process; however, the protective efficacy varied among different BCFA monomers. This indicates that BCFAs can mitigate LPS-induced apoptosis, likely by inhibiting the activity of apoptosis-related genes. Moreover, consistent with our findings, a study by Li Jiao [63] showed that iso-C15:0, anteiso-C15:0, and anteiso-C17:0 alleviated endoplasmic reticulum stress in human hepatocytes induced by palmitic acid. Interestingly, cells co-treated with BCFAs and palmitic acid did not display prominent apoptotic morphology. While palmitic acid treatment resulted in the upregulation of cleaved *Caspase-3* protein levels, cells co-treated with BCFAs did not exhibit this increase, suggesting BCFAs’ protective role against apoptosis [63].

The TOPSIS method has been widely applied in various fields of decision-making and evaluation [64,65,66]. In comparison to other weighting methods [67,68], EWM is straightforward and does not rely on subjective preferences; it uses objective data to determine weights. The Entropy Weight-TOPSIS method is a multi-indicator evaluation technique that fully utilizes original data, unrestricted by sample size or the number of indicators. In our study, the effects of each BCFA monomer on different cellular indicators were inconsistent, indicating limitations in using single factors or primary indicators for evaluation. Therefore, we evaluated the mitigating effects of BCFAs on inflammatory responses in LPS-induced CSIECs based on the Entropy Weight-TOPSIS method. This algorithm addresses the challenge of inconsistent dimensions and measurements by normalizing the factors. The analysis was based on all data collected from our experiments. The results reveal that among the six BCFA monomers, iso-C15:0 demonstrated the most significant effects in CSIECs, while iso-C14:0 exhibited a similarity value close to that of iso-C15:0. This finding suggests that the intestinal characteristics of calves may predispose them to favor the beneficial effects of relatively shorter-chain BCFAs.

## 5. Conclusions

This research indicates that various BCFAs can, to varying degrees, prevent LPS-induced damage by regulating the integrity of cellular tight junction structures as well as genes related to oxidative stress, inflammation, and apoptosis. Among them, iso-C15:0 demonstrates the most effective performance. While this experiment confirmed the beneficial role of BCFAs in alleviating inflammatory responses at the cellular level, the specific targets and mechanisms of action remain unclear. Moreover, cellular experiments have inherent limitations, raising the question of whether these in vitro results will effectively translate to actual production settings. Thus, extensive data from production studies will be necessary for further validation. In the future, iso-C15:0 will be used as the main object for more in-depth research.

## Figures and Tables

**Table 1:** Gene-specific primers for qRT-PCR.

Gene	Primer Sequences (5′−3′)
*IL-1β*	Forward: GCTTCAGGCAGGTGGTGTCGGTCAT
	Reverse: GCGTCACACAGAAACTCGTCGGAGGA
*IL-6*	Forward: CACTGACCTGCTGGAGAAGATGC
	Reverse: CCGAATAGCTCTCAGGCTGAACTG
*IL-8*	Forward: AGCTGGCTGTTGCTCTCTTGG
	Reverse: TGGGGTGGAAAGGTGTGGAATG
*IL-10*	Forward: ACCAGCCACCAATGTTGCTCA
	Reverse: CTTCTCCACCGCCTTGCTCTTG
*TNF-α*	Forward: TGAAGGAAGAGGAGAGGCTCATCG
	Reverse: GTGGTCATCGGAGTTGCTGGTG
*IFN-γ*	Forward: CCGAGCGTGGAGGATCATTGC
	Reverse: CCAACGAGGCACAGCAGGATG
*TLR4*	Forward: GTAAAGAACTTGGAGGAGGGC
	Reverse: TGCTGGGACACCACGACA
*MyD88*	Forward: TATCGGCTGAAGTTGTGCGTGTC
	Reverse: TCAGAGACCACCACCACCATCC
*NF-κB*	Forward: CTCACCGGCCTCACCCTCAC
	Reverse: GGTCCCGCTTCTTTACACACTGG
*ZO-1*	Forward: GAACACGACAGAGCAGCACATAGG
	Reverse: GGCTCGGAGAGGTGGCTAGTG
*Occludin-1*	Forward: GCACGTTCGACCAATGCTCT
	Reverse: CAGGCAAGAGTGGAGGCAAC
*Claudin-1*	Forward: GCTGTGGATGTCCTGCGTGTCR
	Reverse: CCTCGTCGTCTTCCATGCACTTC
*Claudin-4*	Forward: TCATCGGCAGCAACATCGTCAC
	Reverse: CAGCAGCGAGTCGTACACCTTG
*Caspase-3*	Forward: AGACAGTGGTGCTGAGGATGAC
	Reverse: CCAGGTGCTGTAGAATATGCGTAC
*Caspase-8*	Forward: AGCCAGGAGATTGCCAAATGTAAG
	Reverse: TCAGGGTGTCCAAGTTCTCTTCC
*Caspase-9*	Forward: AGTTTGTGGTGGAGGTGAAATGTG
	Reverse: TGACAGCCGTGAGACAGGATG
*BAX*	Forward: TGCTTCAGGGTTTCATCC
	Reverse: CTTCAGACACTCGCTCAG
*BCL-2*	Forward: TGAGTTCGGAGGGGTCATGT
	Reverse: AGGTGCCGGTTCAGGTACTC
*β-actin*	Forward: TGGCGCTTGACTCAGGATTT
	Reverse: CAATCAAGTCCTCGGCCACA

**Table 2:** Oxidative stress-related indicators.

Primary Indicator	Secondary Indicator	Indicator Nature
Oxidative stress-related indicators	ROS	Negative
	SOD	Positive
	GSH	Positive
	GSH-Px	Positive
	CAT	Positive
	MDA	Negative
	T-AOC	Positive

**Table 3:** Energy metabolism-related indicators.

Primary Indicator	Secondary Indicator	Indicator Nature
Energy metabolism-related indicators	MMP	Positive
	Na^+^-K^+^-ATPase	Positive
	Mg^2+^-ATPase	Positive
	Ca^2+^-ATPase	Positive
	Ca^2+^-Mg^2+^-ATPase	Positive
	ATP	Positive

**Table 4:** Indicators related to anti-inflammatory properties.

Primary Indicator	Secondary Indicator	Indicator Nature
Indicators related to anti-inflammatory properties	*IL-1β*	Negative
	*IL-6*	Negative
	*IL-8*	Negative
	*IL-10*	Positive
	*TNF-α*	Negative
	*IFN-γ*	Negative
	*TLR4*	Negative
	*MyD88*	Negative
	*NF-κB*	Negative
	*ZO-1*	Positive
	*Occludin*	Positive
	*Claudin-1*	Positive
	*Claudin-4*	Positive

**Table 5:** Apoptosis-related indicators.

Primary Indicator	Secondary Indicator	Indicator Nature
Apoptosis-related indicators	*Caspase-3*	Negative
	*Caspase-8*	Negative
	*Caspase-9*	Negative
	*BAX*	Negative
	*BCL-2*	Positive

**Table 6:** Data standardization of processing results.

Primary Indicator	Indicator Name	iso-C14:0 + LPS	iso-C15:0 + LPS	iso-C16:0 + LPS	iso-C17:0 + LPS	Anteiso-C15:0 + LPS	Anteiso-C17:0 + LPS
Oxidative stress-related indicators	ROS	0.7937	1.0001	0.9056	0.0001	0.2537	0.6742
	SOD	1.0001	0.6291	0.1269	0.4007	0.1953	0.0001
	GSH	1.0001	0.7262	0.7090	0.5155	0.1730	0.0001
	GSH-Px	0.9414	1.0001	0.0001	0.3355	0.7895	0.6669
	CAT	1.0001	0.9340	0.3950	0.0001	0.0960	0.3019
	MDA	1.0001	0.2673	0.4025	0.1324	0.0001	0.2169
	T-AOC	0.8467	0.7532	1.0001	0.5994	0.0001	0.1625
Energy metabolism-related indicators	MMP	1.0001	0.3838	0.9443	0.1938	0.8510	0.0001
	Na^+^-K^+^-ATPase	0.8847	0.9521	0.0001	0.1047	0.4825	1.0001
	Mg^2+^-ATPase	1.0001	0.4349	0.5278	0.4877	0.0001	0.5298
	Ca^2+^-ATPase	1.0001	0.8386	0.9967	0.4786	0.0001	0.3192
	Ca^2+^-Mg^2+^-ATPase	0.1784	0.2845	0.0001	0.2112	1.0001	0.3684
	ATP	1.0001	0.3705	0.4021	0.0001	0.2453	0.1518
Indicators related to anti-inflammatory properties	*IL-1β*	1.0001	0.8598	0.6834	0.0001	0.2683	0.3720
	*IL-6*	0.0358	1.0001	0.5967	0.0001	0.0504	0.2161
	*IL-8*	0.3693	1.0001	0.6989	0.0358	0.0001	0.6017
	*IL-10*	0.6522	0.0001	0.2200	0.8314	1.0001	0.1487
	*TNF-α*	1.0001	0.3126	0.3753	0.0001	0.5881	0.4746
	*IFN-γ*	0.3211	1.0001	0.8583	0.0001	0.1919	0.4524
	*TLR4*	0.2108	1.0001	0.7461	0.0001	0.1080	0.3556
	*MyD88*	1.0001	0.9801	0.4891	0.0001	0.8517	0.8311
	*NF-κB*	1.0001	0.2587	0.3938	0.0001	0.8993	0.1182
	*ZO-1*	0.6603	0.0143	0.0001	0.6142	1.0001	0.3339
	*Occludin*	0.5421	0.0001	0.1145	1.0001	0.9120	0.2780
	*Claudin-1*	1.0001	0.0001	0.4367	0.9532	0.6975	0.3804
	*Claudin-4*	0.0001	0.6179	0.4209	1.0001	0.4301	0.6384
Apoptosis-related indicators	*Caspase-3*	0.0217	0.5309	1.0001	0.0001	0.0769	0.1665
	*Caspase-8*	1.0001	0.8593	0.3550	0.0001	0.8074	0.5291
	*Caspase-9*	1.0001	0.8025	0.7539	0.0001	0.3900	0.5512
	*BAX*	1.0001	0.2971	0.8716	0.0001	0.3628	0.2671
	*BCL-2*	0.4735	0.4256	0.0001	0.4757	1.0001	0.0708

**Table 7:** Entropy weight assignment results for various indicators.

Primary Indicator	Indicator Name	Entropy	Weight	Positive Ideal Solution	Negative Ideal Solution
Oxidative stress-related indicators	ROS	0.8557	0.0237	0.0237	0.0001
	SOD	0.7715	0.0375	0.0375	0.0001
	GSH	0.8362	0.0269	0.0269	0.0001
	GSH-Px	0.8669	0.0219	0.0219	0.0001
	CAT	0.7683	0.0381	0.0381	0.0001
	MDA	0.7568	0.0400	0.0400	0.0001
	T-AOC	0.8356	0.0270	0.0270	0.0001
Energy metabolism-related indicators	MMP	0.8238	0.0290	0.0290	0.0001
	Na^+^-K^+^-ATPase	0.8082	0.0315	0.0315	0.0001
	Mg^2+^-ATPase	0.8692	0.0215	0.0215	0.0001
	Ca^2+^-ATPase	0.8535	0.0241	0.0241	0.0001
	Ca^2+^-Mg^2+^-ATPase	0.7708	0.0377	0.0377	0.0001
	ATP	0.7837	0.0355	0.0356	0.0001
Indicators related to anti-inflammatory properties	*IL-1β*	0.8412	0.0261	0.0261	0.0001
	*IL-6*	0.6253	0.0616	0.0616	0.0001
	*IL-8*	0.7709	0.0376	0.0377	0.0001
	*IL-10*	0.7904	0.0344	0.0345	0.0001
	*TNF-α*	0.8484	0.0249	0.0249	0.0001
	*IFN-γ*	0.8111	0.0310	0.0310	0.0001
	*TLR4*	0.7598	0.0395	0.0395	0.0001
	*MyD88*	0.8834	0.0192	0.0192	0.0001
	*NF-κB*	0.7709	0.0377	0.0377	0.0001
	*ZO-1*	0.7512	0.0409	0.0409	0.0001
	*Occludin*	0.7840	0.0355	0.0355	0.0001
	*Claudin-1*	0.8594	0.0231	0.0231	0.0001
	*Claudin-4*	0.8684	0.0216	0.0216	0.0001
Apoptosis-related indicators	*Caspase-3*	0.6115	0.0638	0.0639	0.0001
	*Caspase-8*	0.8657	0.0221	0.0221	0.0001
	*Caspase-9*	0.8721	0.0210	0.0210	0.0001
	*BAX*	0.8140	0.0306	0.0306	0.0001
	*BCL-2*	0.7865	0.0351	0.0351	0.0001

**Table 8:** The closeness values and ranking of indicators.

Ranking Order	Overall Indicators		Oxidative Stress		Energy Metabolism		Anti-Inflammatory Property		Apoptosis	
	Closeness Values	BCFA	Closeness Values	BCFA	Closeness Values	BCFA	Closeness Values	BCFA	Closeness Values	BCFA
1	0.5579	iso-C15:0	0.9249	iso-C14:0	0.6690	iso-C14:0	0.5545	iso-C15:0	0.6502	iso-C16:0
2	0.5535	iso-C14:0	0.6435	iso-C15:0	0.5164	anteiso-C15:0	0.4798	iso-C14:0	0.5197	iso-C15:0
3	0.5071	iso-C16:0	0.4654	iso-C16:0	0.5068	iso-C15:0	0.4774	iso-C16:0	0.4153	iso-C14:0
4	0.4281	anteiso-C15:0	0.2983	iso-C17:0	0.4275	iso-C16:0	0.4735	anteiso-C15:0	0.3978	anteiso-C15:0
5	0.3310	anteiso-C17:0	0.2833	anteiso-C17:0	0.4196	anteiso-C17:0	0.3659	iso-C17:0	0.2396	anteiso-C17:0
6	0.3090	iso-C17:0	0.2210	anteiso-C15:0	0.2331	iso-C17:0	0.3486	anteiso-C17:0	0.1739	iso-C17:0

## Data Availability

The data will be made available upon request.
